# The horse as a natural model to study reproductive aging-induced aneuploidy and weakened centromeric cohesion in oocytes

**DOI:** 10.18632/aging.104159

**Published:** 2020-11-02

**Authors:** Marilena Rizzo, Nikola du Preez, Kaatje D. Ducheyne, Claudia Deelen, Mabel M. Beitsma, Tom A. E. Stout, Marta de Ruijter-Villani

**Affiliations:** 1 Department of Clinical Sciences, Faculty of Veterinary Medicine, Utrecht University, Utrecht, 3584 CM, the Netherlands; 2 Sussex Equine Hospital, Ashington, RH20 3BB, United Kingdom; 3 Department of Production Animal Studies, University of Pretoria, Pretoria, 0110, South Africa

**Keywords:** oocyte, aging, aneuploidy, cohesion, mare

## Abstract

Aneuploidy of meiotic origin is a major contributor to age-related subfertility and an increased risk of miscarriage in women. Although age-related aneuploidy has been studied in rodents, the mare may be a more appropriate animal model to study reproductive aging. Similar to women, aged mares show reduced fertility and an increased incidence of early pregnancy loss; however, it is not known whether aging predisposes to aneuploidy in equine oocytes. We evaluated the effect of advanced mare age on (1) gene expression for cohesin components, (2) incidence of aneuploidy and (3) chromosome centromere cohesion (measured as the distance between sister kinetochores) in oocytes matured *in vitro*. Oocytes from aged mares showed reduced gene expression for the centromere cohesion stabilizing protein, Shugoshin 1. Moreover, *in vitro* matured oocytes from aged mares showed a higher incidence of aneuploidy and premature sister chromatid separation, and weakened centromeric cohesion. We therefore propose the mare as a valid model for studying effects of aging on centromeric cohesion; cohesion loss predisposes to disintegration of bivalents and premature separation of sister chromatids during the first meiotic division, leading to embryonic aneuploidy; this probably contributes to the reduced fertility and increased incidence of pregnancy loss observed in aged mares.

## INTRODUCTION

Advanced age in women predisposes to chromosome segregation errors during the meiotic divisions in oocytes. When this happens, the resulting oocyte will have an abnormal number of chromosomes (aneuploidy) and, once fertilized, will give rise to an aneuploid embryo. Embryonic aneuploidy is known to be the major cause of developmental arrest, implantation failure, miscarriage and congenital birth defects in human reproduction [1, 2]. While 20% of mature oocytes in women between the ages of 25 and 30 years exhibit aneuploidy (i.e. an abnormal number of chromosomes), in women older than 35 years the proportion of aneuploid oocytes increases to 50%; then rises again to more than 60% for women above 40 years old [3]. However, studies on human oocytes and embryos are limited by availability, practicality and, above all, ethical concerns; and although non-human primates might appear to represent the closest animal model [4], some of the aforementioned limitations also apply. However, women are not the only mammals in which fertility is susceptible to the effects of aging. Age-related aneuploidy and variations in fertility have been investigated in various animal models, with the majority of targeted studies carried out on rodents. Despite the considerable advantages of rodent models, there are also important differences in reproductive physiology and lifespan between mice and women. By contrast, in horses just as in women, advanced female age is associated with decreased fertility and an increased risk of early pregnancy loss [5–7]. The likelihood of an equine embryo developing successfully to day 4 decreases in mares above 14 years of age (81% vs 96%) [8] while mares older than 18 years are three times more likely to suffer pregnancy loss between Days 16 and 60 than mares below 12 years of age (30% vs 10%) [9–11]. Analogies between mares and women are however not limited to the decrease in fertility with increasing age, but can be extended to other aspects of their reproductive biology: women and mares are both (1) mono-ovulatory, with a long follicular phase and a similar time course to ovulation; (2) show comparable age-related changes in cycle length and hormone concentrations; (3) have a long time interval (decades) to reproductive senescence; (4) exhibit meiotic oocyte arrest for decades; (5) show reduced fertility as a result of intrinsic oocyte defects [5, 12–14]. We recently showed that advanced maternal age impairs the ability of horse oocytes to correctly align their chromosomes on the metaphase plate [15], presumably predisposing them to aneuploidy. Even though it has previously been argued that the mare represents a potentially valuable model for reproductive aging in women [5], the incidence and the origin of aneuploidy in horse oocytes is unknown. Although various factors may contribute to the genesis of chromosome segregation errors and consequently to aneuploidy, in human and murine oocytes weakened cohesion between the centromeres is proposed to play a pivotal role [16–19]. During the first meiotic division, the homologous chromosomes are held together by both recombination sites and cohesin complexes. In mammalian oocytes, the cohesin complex is a ring-like protein structure localized along the chromosome arms and centromeres and is composed of two maintenance proteins (Smc1β and Smc3), a kleisin (Rec8) and Stag3 [17]. Other accessory proteins, such as Pds5B, Sororin and Wapl are weakly associated with the cohesin complex and regulate the dynamic interaction between cohesin and the chromatin. In this respect, Sororin and Pds5B interact to stabilize cohesin loading onto the chromatin, whereas Wapl facilitates dissociation of cohesin by competing with Sororin for binding to Pds5B [17].

During anaphase of the first meiotic division (anaphase I), Separase cleaves the cohesin along the chromosome arms, allowing bivalent resolution and the consequent release and separation of the homologous chromosomes [18]. The centromeric cohesin is protected against Separase by Shugoshin and continues to hold the sister chromatids together during the initial stages of the second meiotic division. At the onset of anaphase II, Shugoshin re-localizes from centromeric chromatin to the kinetochores, allowing the residual centromeric cohesin to be cleaved by Separase, which in turn allows segregation of the sister chromatids [17].

Although components of the cohesin complex are loaded onto the chromosomes during the pre-meiotic S phase of fetal development, in adult women and aged mice a gradual loss of cohesin has been shown to occur in oocytes as a result of aging [16, 19, 20]. This can lead to the premature separation of the two sister chromatids of a bivalent, generating two univalents or even four separate single chromatids. In the present study, we investigate the suitability of the mare as an animal model for future studies of the effect of advanced maternal age on the stability of centromeric cohesion and the risk of oocyte aneuploidy.

## RESULTS

One thousand-one hundred and seventy-one cumulus-oocyte complexes (COCs) were collected from the ovaries of 221 mares (106 young and 115 old mares). Mare ages ranged between 2 and 14 years (mean ± SD: 9.4 ± 3.0 years) for the young group, and between 16 and 27 years (mean ± SD: 20.5 ± 3.6 years) for the old group. No significant difference between the young and old groups was observed for the success of first polar body (PB) extrusion after *in vitro* maturation (319/641, 49.8 % vs 241/530, 45.5 %; P = 0.5).

For the gene expression study, three hundred and twenty oocytes were divided into 8 groups on the basis of mare age (young versus old), cumulus appearance before maturation (compact versus expanded) and extrusion of the first polar body after maturation (MII and non-MII). For each of the 8 groups, 4 pools of 10 oocytes were used for mRNA extraction.

**Figure 1:**
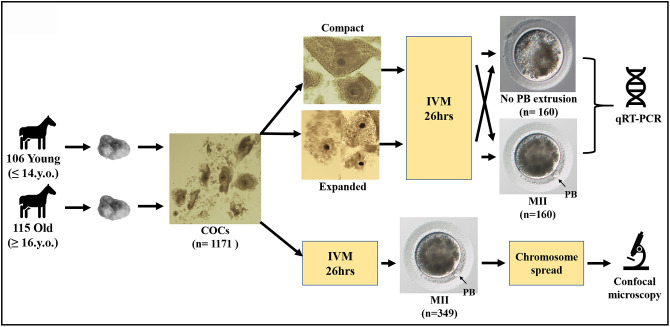
**Schematic representation of the study design.**

Three hundred and forty-nine oocytes that showed first polar body extrusion after *in vitro* maturation were used to make chromosome spreads. However, a usable chromosome spread that could be imaged resulted in only 199 cases (the rest of the oocytes was lost or damaged during handling); moreover, 131 of these spreads could not be analyzed fully due to either insufficient separation of the chromosomes or a poor fluorescent signal for the kinetochore stain. Only spreads where all of the sister chromatids and kinetochores were distinguishable were used for the analysis. Single or unpaired sister chromatids were excluded from interkinetochore distance analysis. A schematic representation of the study design, together with images of equine oocytes with or without polar body, and an expanded or compact surrounding cumulus, are shown in Figure 1.

### MII oocytes from old mares show an increased incidence of aneuploidy

**Figure 2:**
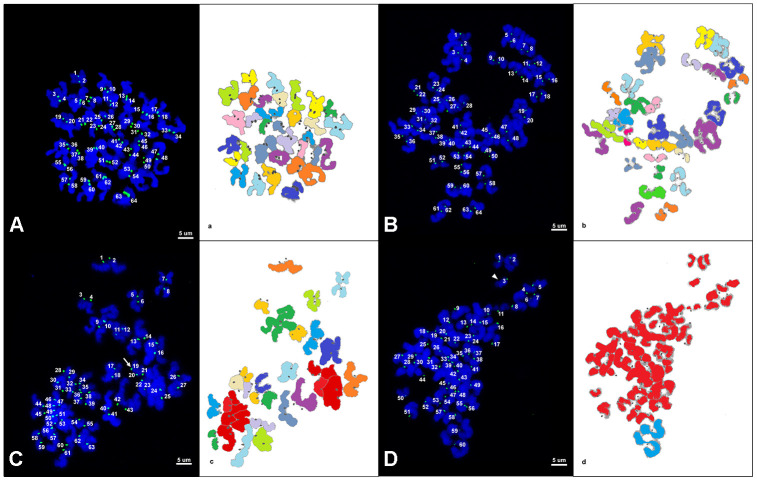
****(**A**–**D**) Representative maximum projection of chromosome spreads for euploid and aneuploid MII oocytes from young and old mares. Green, kinetochores (CREST); blue, chromatin (Hoechst). (**a**–**d**) explanatory drawing of the chromosome spreads. Different sister chromatid pairs have different colors. For the chromatids colored in red, it was not possible to distinguish single pairs and they were therefore used only for ploidy assessment and not for interkinetocore distance measurement. A total of 64 CREST positive foci (32 pairs of sister chromatid kinetochores) are shown in an euploid MII oocyte from a young mare (**A**) and an old mare (**B**). (**C**) A total of 63 CREST positive foci are displayed in an aneuploid MII oocyte from an old mare; the white arrow indicates an uneven and unpaired kinetochore. (**D**) A total of 60 CREST positive foci are seen in an aneuploid MII oocyte from an old mare; the arrow head indicates an unpaired kinetochore. Bar, 5μm.

**Figure 3:**
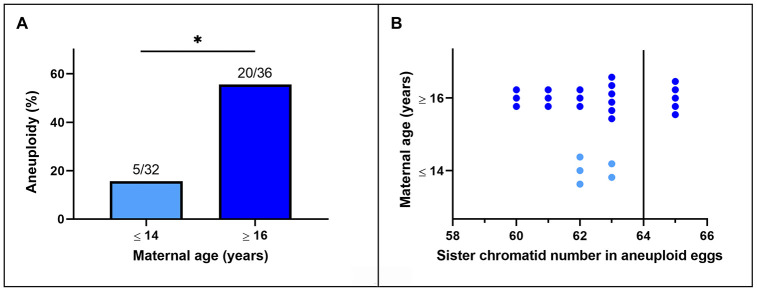
****(**A**) Incidence of aneuploidy in *in vitro* matured MII horse oocytes from mares of different ages (*, p< 0.05). Numbers above bars indicate the number of aneuploid oocytes as a proportion of oocytes analyzed. (**B**) Frequency distribution of sister chromatid counts for the oocytes identified as aneuploid. The vertical line indicates the euploid number.

Representative images of chromosome spreads for euploid and aneuploid oocytes from young and old mares are shown in Figure 2A–2D. MII oocytes from old mares showed a significantly higher incidence of aneuploidy (20/36 = 55.6 %) than oocytes from young mares (5/32 = 15.6 %) (Figure 3A; p < 0.05).

Of the total of 25 aneuploid oocytes from young (5) and old (20) mares, 20 (80%; 5 and 15 from young and old mares respectively) showed hypoploidy (<64 sister chromatids). In the aged mare group, 14 of the 20 aneuploid oocytes (70%) showed an uneven number of sister chromatids (Figures 3B and 2C), which is consistent with unbalanced premature separation of sister chromatids (PSSC) during meiosis I. In the young group, the incidence of even (3/5) and uneven (2/5) numbers of sister chromatids in aneuploid oocytes was similar. In addition, one euploid and one aneuploid oocyte from the old mare group and one aneuploid oocyte from the young mare group showed 2 unpaired sister chromatids (Figure 2D).

### MII oocytes from old mares show weakened centromeric cohesion

**Figure 4:**
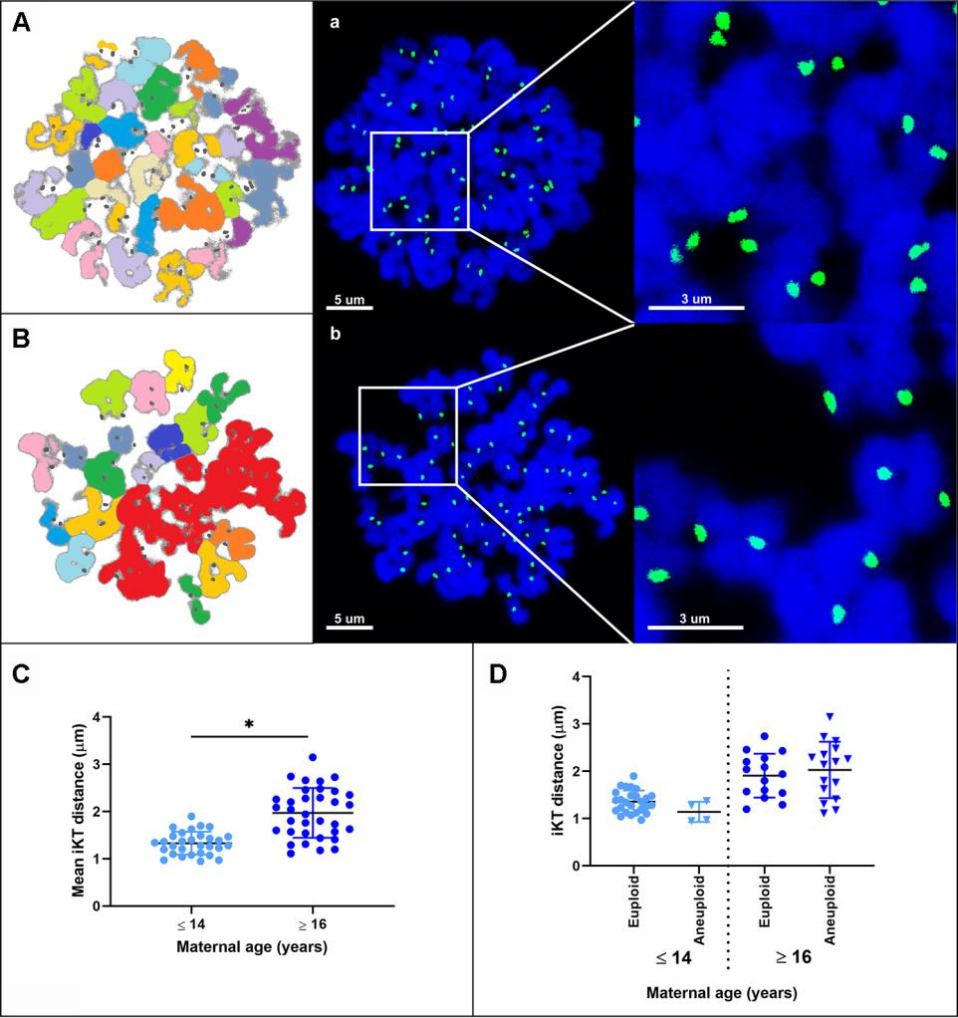
****Representative maximum projection images of chromosome spreads for *in vitro* matured MII oocytes from young (**A**) and aged (**B**) mares. Green, kinetochores (CREST); blue, chromatin (Hoechst 33342). (**A**, **B**) explanatory drawing of the chromosome spreads. Different sister chromatid pairs have different colors. Note the increased interkinetochore distance (i.e. separation of the CREST signals) in oocytes from old mares. Bar, 5 and 3 μm. Scatterplots of interkinetochore distance categorized by mare age (**C**) (*, p < 0.0001) and by mare age and aneuploidy (**D**).

The interkinetochore distance between sister chromatids was greater in MII oocytes from old mares (mean ± SD, 1.96 ± 0.74 μm) than in those from young mares (1.33 ± 0.40 μm; p < 0.0001) (Figure 4A–4C). When oocytes were divided on the basis of ploidy, the interkinetochore distance was similar between euploid and aneuploid oocytes within young (mean ± SD, 1.32 ± 0.32 vs. 1.14 ± 0.18 μm) or within old mares (mean ± SD, 1.91 ± 0.44 vs. 2.03 ± 0.57 μm), respectively (Figure 4D).

### MII oocytes from old mares show reduced expression of *Shugoshin 1* mRNA

**Figure 5:**
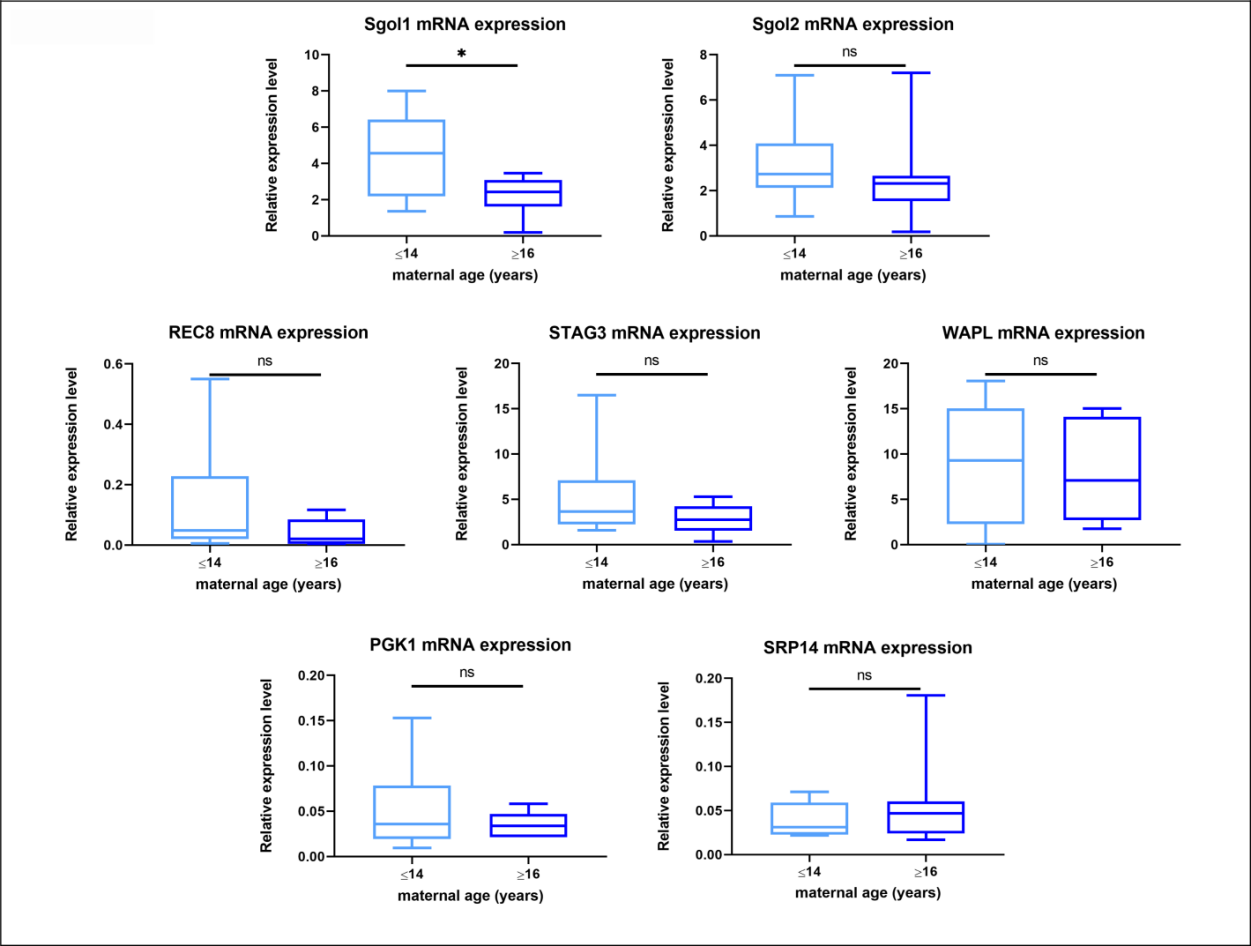
**Box plot showing mRNA expression for housekeeping genes and target genes in oocytes from young (≤14 years) and old (≥16 years) mares.** The boxes show the interquartile range, with the median value indicated by the horizontal line; whiskers show the range. *P < 0.05.

Quantitative RT-PCR revealed measurable mRNA expression for all of the target genes in all samples (Supplementary Figure 1), while amplification of the -RT blanks did not result in measurable amounts of product. No difference in the gene expression for Rec8, Stag3, Sgo2 and Wapl was found in oocytes from young and old mares, whereas Sgo1 expression was significantly lower in oocytes from old compared to those from young mares, irrespective of the success of maturation or initial cumulus appearance (Figure 5).

## DISCUSSION

The results of this study demonstrate that advanced age predisposes to aneuploidy in mare oocytes, as has previously been reported for women [3]. Not only was the incidence of aneuploidy in MII oocytes from mares (about 15% in young and 55% in old mares) comparable to that reported for women (20% in women younger than 35 years; 60% in women older than 35 years) [3], but the underlying mechanisms of chromosome mis-segregation also appear to be similar. It has been reported that 80% of aneuploidies seen in the oocytes of reproductively aged women are the result of premature separation of the sister chromatids [3, 21–23], and that cohesion weakening along both the chromosome arms and centromeric regions are responsible for the elevated incidence of PSSC in older women [23]. Whereas in mice chromatid count in aneuploid oocytes is biased to even numbers [24] and only 31% are consistent with age-related PSSC [25, 26], our results show that 70% of aneuploid oocytes from aged mares have an uneven chromatid number. This is most likely the result of premature separation of the bivalents followed by random segregation of the sister chromatids during the first meiotic division. This is consistent with weakening of centromeric cohesion as further evidenced by the increased distance between sister kinetochores in MII oocytes from aged mares. It is interesting to note that the age-related increase in interkinetochore distance observed in mare MII oocytes (1.33 ± 0.40 μm in young compared to 1.96 ± 0.74 μm in aged mares) is of a similar magnitude to that identified in women (0.82 ± 0.03 μm in women <35 years, compared to 1.1 ± 0.03 μm in women > 35 years [16]). Unlike in mice, where the interkinetochore distance is increased not only in oocytes from aged females but also in aneuploid oocytes from young females [26], the interkinetochore distance did not differ between euploid and aneuploid MII equine oocytes within a given mare age group, suggesting that mare oocytes are a better model for studying the effect of maternal aging on centromeric cohesion. The similarities in the incidence of age-related oocyte aneuploidy and the centromeric cohesion weakening between mares and women identified in our study, together with other important similarities previously described between these two species in reproductive biological characteristics and reproductive senescence [5], indicate that the mare is a useful model for studying the age-dependent mechanisms that predispose to oocyte and embryo aneuploidy.

The molecular origin of the observed increase in interkinetochore distance remains to be elucidated. It may involve the loss of a particular component of the cohesion complex. The maintenance and regulation of cohesion along the chromosome arms and centromeres is important to avoid premature breakdown of the bivalent into two separate univalents and precocious separation of sister chromatids. It is generally accepted that the protection of cohesion by the Shugoshin protein family is a mechanism conserved for mitosis and meiosis, and both members of the Shugoshin protein family (Sgo1 and Sgo2) have been reported in mammals [27–30]. While it has been clearly shown that localisation of Shugoshin 2 (Sgo2) to the centromere mediates cohesin protection during meiosis I in mouse oocytes [31, 32] and that Sgo2 depletion is sufficient to cause loss of centromeric cohesion in mouse oocytes despite the presence of Shugoshin 1 (Sgo1), the exact role of Sgo1 in meiosis is unclear. Recently a possible role of Shugoshin 1 in the prevention of premature separation of sister chromatids during meiosis I was proposed [33, 34]. It is therefore possible that the reduced expression of *Shugoshin 1* mRNA observed in aged mare oocytes reflects a reduction of Shugoshin 1 function during meiosis, which could contribute to deterioration of centromeric cohesion in oocytes.

Another possible cause of centromeric cohesion loss in oocytes from older females is increased oxidative damage [35, 36]. During *in vitro* maturation, oocytes are subject to various oxidative insults; since oocytes from aged mares are known to be subject to mitochondrial damage [37, 38] and therefore oxidative stress during *in vitro* maturation, it is possible that the higher incidence of aneuploidy and weakened centromere cohesion observed in the present study are higher than they may be for oocytes of aged mares allowed to mature *in vivo*.

**Figure 6:**
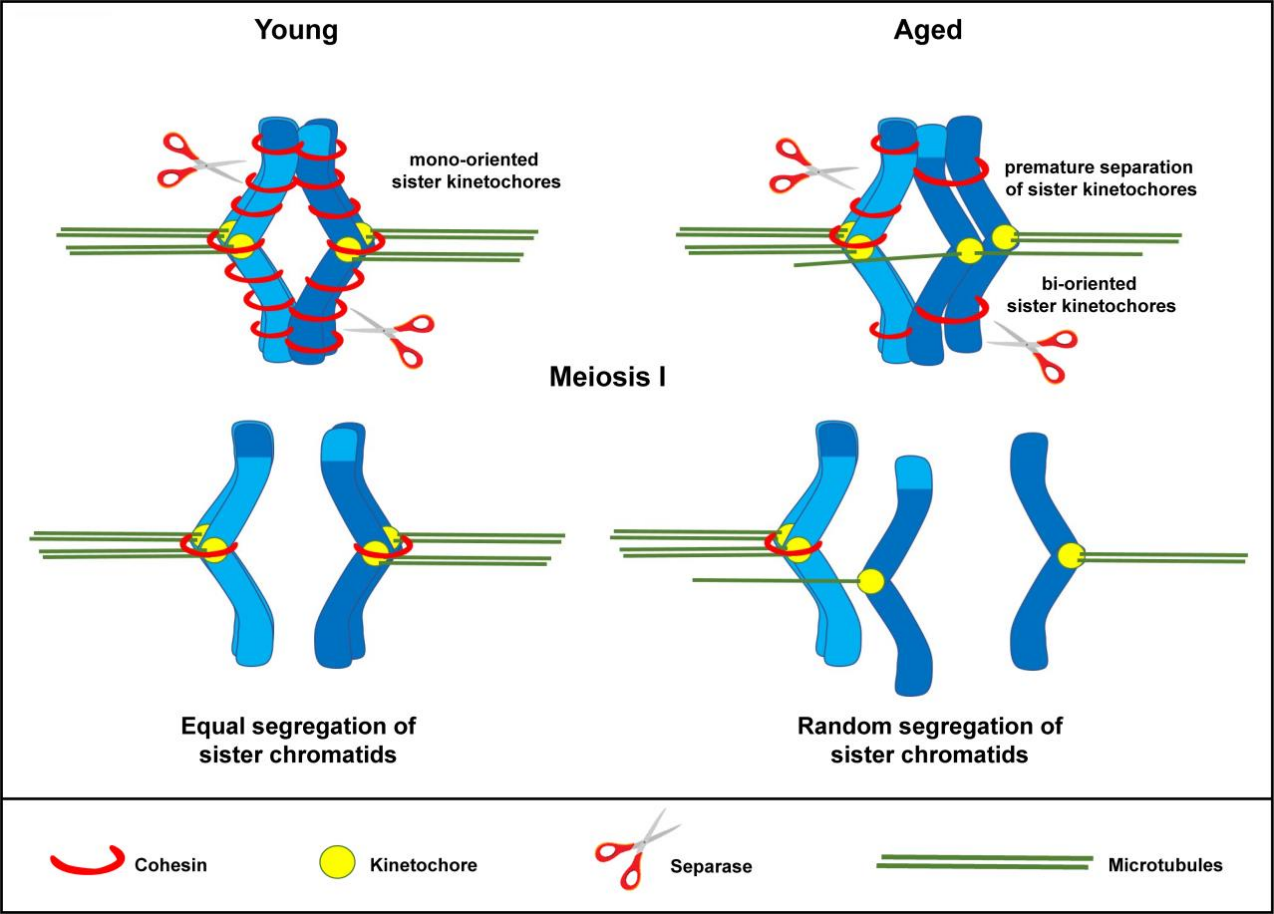
**Schematic representation for loss of chromosome cohesion and the generation of single chromatids in MII oocytes as a result of maternal aging.** The effect of advanced maternal age is depicted on a bivalent (dark and light blue) during meiosis I. Aging is associated with weakened cohesion (red rings). The increased distance between sister chromatid kinetochores prevents them from working as a single unit. They are therefore subject to random segregation, as opposed to equal segregation.

In conclusion, we propose that similar to what has been observed in murine and human oocytes [19, 20], the weakening of centromeric cohesion observed in *in vitro* matured oocytes from aged mares may predispose to premature separation of the sister chromatids during the first meiotic division. As a result, the sister chromatids could prematurely segregate during the first meiotic division, either in a balanced or unbalanced fashion (Figure 6), predisposing to embryonic aneuploidy of meiotic origin. This may partly explain the reduced fertility and the increased incidence of early pregnancy loss in aged mares, and suggests that the horse could be a valuable animal model for studying the molecular mechanisms underlying the effects of maternal aging on oocyte chromosome mis-segregation and aneuploidy. On the other hand, because of the lack of specific research tools developed for the horse and the inefficiency and high costs of producing genetically modified horses for experimental purposes, mare oocytes are unlikely to provide the mechanistic depth possible for research with mouse oocytes; we do not therefore propose that the horse would substitute for rodents as an animal model, but rather that studies on horse material could complement and strengthen rodent studies for translational purposes, thereby accepting that no single animal model can mimic all features of human oocyte senescence.

## MATERIALS AND METHODS

### Oocyte collection and culture

Ovaries were recovered from slaughtered mares within 15 min after death, divided into two groups depending on the age of the mare (young, ≤ 14 years; old, ≥ 16 years) and maintained at 21-28° C for 2-4 hours during transport, as described previously [15]. Mare age was determined by reading the microchip and consulting the corresponding passport. Cumulus-oocyte complexes (COCs) were collected by scraping the wall of incised follicles with a bone curette and flushing out the dislodged cells with embryo flushing medium (Euroflush; IMV Technologies, Leeuwarden, The Netherlands) supplemented with 0.4 % Heparin (Heparin sodium 5000 IU/mL; LEO Pharma BV, Denmark) as described previously [15]. To prevent the collection of *in vivo* matured oocytes, only follicles ≤30 mm were scraped. The collected COCs were identified using a dissecting microscope and only COCs with at least one layer of intact cumulus cells were used in the following studies. Collected COCs were first held at 21–22° C for 12 hours in HEPES-buffered synthetic oviduct fluid (H-SOF; Avantea, Italy) and subsequently matured *in vitro* for 26 hours at 38.5° C in 5% CO_2_-in-air in a 50:50 mixture of Dulbecco’s minimal essential medium (DMEM) and Ham’s F12 (GIBCO BRL Life Technologies, Bleiswijk, The Netherlands) supplemented with 10% fetal calf serum (Sigma-Aldrich Chemical Co., St. Louis, Missouri, USA), 0.125 μg/mL epidermal growth factor (Peprotech Inc., Rocky Hill, New Jersey, USA), 0.1 IU/mL follicle-stimulating hormone, 0.6 mmol/L cysteine and 0.1 mmol/L cysteamine (Sigma-Aldrich Chemical Co.), 0.1% insulin, 0.1% transferrin and 0.1% sodium selenite (VWR International BV, Amsterdam, The Netherlands). After oocyte maturation, cumulus cells were removed by exposing the COCs briefly to H-SOF supplemented with 1 μg/mL hyaluronidase (Sigma-Aldrich Chemical Co.) before gentle pipetting through 131 and 55 μm pipettes (EZ-strip, Research Instruments Ltd, Falmouth, UK). For the gene expression study, the oocytes were further divided on the base of the appearance of the COCs (expanded or compact) before maturation and on the successful extrusion of the first polar body after maturation. Only oocytes showing first polar body extrusion were used for producing chromosome spreads.

### RNA extraction and cDNA synthesis

Total RNA was extracted from pools of 10 oocytes using the AllPrep DNA/RNA/Protein Mini kit (Qiagen, Venlo, The Netherlands) following the manufacturer’s instructions. The quantity and quality of total RNA were determined, respectively, by spectrophotometry and using an Agilent BioAnalyzer 2100 (Agilent, Palo Alto, California, USA) with an RNA 6000 Nano Labchip kit (Agilent) in accordance with the manufacturer’s instructions. Only samples with an RNA integrity number (RIN) of 7.5 or greater were used for analysis. Reverse transcription was performed as described previously [39] using Superscript III (Invitrogen Corporation, Carlsbad, California, USA), in a total volume of 20 μl made up of 10 μl of sample containing 1000 ng of RNA which had been treated with DNAse I (30 min at 37° C followed by 10 min at 65° C; 1 IU/mg of RNA; RNAse-Free DNase set, Qiagen).

### Quantitative RT-PCR

Quantitative RT-PCR was performed as described previously [39]. The primers used in the present study (Table 1) were produced at Eurogentec (Seraing, Belgium), with specificity tested by DNA sequencing (ABI PRISM 310 Genetic analyzer; Applied Bio-system, Foster City, California, USA). Real-time PCR was carried out in 15 μl of reaction mix including 7.5 μl of IQ SYBR® Green Supermix (BioRad, Veenendaal, The Netherlands), 0.5 μM of primer, and 1 μl of cDNA, on an IQ5 Real-Time PCR detection System (BioRad). Cycle conditions included a 3 min denaturation step at 95° C, followed by 40 cycles of amplification (15 s at 95° C, 30 s at the primer specific annealing temperature and 30 s at 72° C). A melting curve and standard curve were performed to verify product specificity and enable quantification of expression for each gene. Relative gene expression was expressed as the ratio of target gene expression to the geometric mean of expression for two housekeeping genes (PGK1 and SRP14), selected after stability evaluation using GeNorm (Biogazelle, Zwijnaarde, Belgium).

**Table 1:** Details of primer pairs used in the present study.

**Gene**	**Sequence**	**T_a_ (°C)**	**Amplicon size (bp)**	**GenBank Accession no.**
**REC8**	F: 5'-GGTCTACTTTCAACAATGCCAG-3'	55°	100	XM_005603388.1
	R: 5'-GCTCCACCATATCAATGCGG-3'			
**SGOL1**	F: 5'-CAGGGATTTATTTGTGACGG-3'	63°	115	XM_001917735.3
	R: 5'-TGTCTTGATTAGGAATGGTAGG-3'			
**SGOL2**	F: 5'-GATATACTTCCCGAAGAAAGCC-3'	57°	160	XM_005601672.1
	R: 5'-TTTGATTCCCGAGATGATACAC-3'			
**STAG3**	F: 5'-CTATGACACTAATGACCTCCCT-3'	57°	280	XR_288160.1
	R: 5'-CATCCAACACCCAATCTCCT-3'			
**WAPL**	F: 5'-AAATCAAGAGTTCACTGACGAC-3'	60°	207	XM_023648739.1
	R: 5'-ACAAAGGGACAAATTCTGATGG-3'			
**PGK1**	F: 5'-CTGTGGGTGTATTTGAATGG-3'	54°	151	XM_005614287.1
	R: 5'-GACTTTATCCTCCGTGTTCC-3'			
**SRP14**	F: 5'-CTGAAGAAGTATGACGGTCG-3'	55°	101	XM_001503583.3
	R: 5'-CCATCAGTAGCTCTCAACAG-3'			

### Chromosome spreads and immunostaining

The method used to obtain chromosome spreads is adapted from Silva et al 2018 [40]. In short, the zona pellucida was removed by exposing the oocytes briefly to 0.1% pronase (Sigma-Aldrich Chemical Co.) in a phosphate-buffered saline-polyvinylpyrrolidone (PBS-PVP) solution (B. Braun, Hessen, Germany - Sigma-Aldrich Chemical Co.). After inducing cell swelling by incubating the oocytes in a hypotonic solution consisting of 1% sodium citrate (VWR International BV) in ultrapure water (Milli-Q®, MQ) for 8 minutes, the oocytes were lysed as described previously [41], using a chromosome spread solution consisting of 0.15% Triton X-100 and 3 mM dithiothreitol (Sigma-Aldrich Chemical Co.), and 0.2% paraformaldehyde (Electron Microscopy Sciences, Hatfield, Pennsylvania, USA) on a cover slip. After lysis, air was gently blown on the slide using a plastic Pasteur pipet (Sigma-Aldrich Chemical Co.) to evenly spread the chromosomes, after which the slides were air-dried overnight in a humidified chamber at 37° C. Prior to staining of chromosome spreads, non-specific staining was blocked by incubation for one hour in 3% bovine serum albumin (BSA, Sigma-Aldrich Chemical Co.) in PBS at room temperature. The chromosome spreads were then incubated overnight at 4° C in PBS containing a 1:250 dilution of a purified human anti-centromere CREST antibody (Cat. No. 15-235; Antibodies Incorporated, Davis, California, USA) and 0.5% BSA. The chromosome spreads were then washed three times in PBS with 5% BSA (PBS-BSA) before being incubated in PBS-BSA containing a 1:100 dilution of goat anti-human Alexa Fluor 488 (A11013; Life Technologies, Eugene, Oregon, USA) for 3 hours at room temperature. After three washes with 0.1% Triton X-100 in PBS-BSA, the chromosome spreads were incubated in MQ containing a 1:1000 dilution of Hoechst 33342 (Sigma-Aldrich Chemical Co.) for 10 minutes at room temperature to stain the chromatin. After an additional washing step with MQ, the coverslips were mounted on glass slides with Vectashield H-1000 (Vector Laboratories Inc., Peterborough, UK).

### Image acquisition and analysis

Image acquisition was performed using a confocal laser scanning microscope (Leica TCS-SPE-II; Leica Microsystems, Wetzlar, Germany) equipped with a 63x objective. Hoechst 33342 was stimulated with a 405 nm laser and the emission was detected between 414 and 466 nm (blue channel), Alexa Fluor 488 was separately stimulated with a 488 nm laser and emission was detected in the 511-577 nm range (green channel). A 3-dimensional image of the chromosomes and the kinetochores was acquired using sequential confocal sections (Z-stacks) at 0.17 μm intervals and then analyzed using Imaris 8.2 software (Bitplane AG, Zurich, Switzerland). Euploid MII horse oocytes should contain a total of 32 dyads, composed of 64 sister chromatids, with two sister kinetochores on each sister chromatid pair. Aneuploidy results in deviation from this number. The Imaris spot tool was used to segment the kinetochores, by selecting the green channel, applying a Gaussian smoothing filter (detail level of 0.0853 μm) and a threshold for surface creation on the basis of absolute intensity (using 51 and 166 arbitrary units as lower and upper thresholds, respectively). The distances between sister chromatids (interkinetochore distance) was measured using the MatLab (Math Works, Natick, Massachusetts, USA) plug-in function “spots-to-spots closest distance”. This automatic sister kinetochore recognition was checked for accuracy and manually adjusted when kinetochores were falsely linked.

### Statistical analysis

All statistical analysis was performed using GraphPad Prism 8.0 software (GraphPad Software, San Diego, California, USA). Normality was analyzed using the D’Agostino-Pearson and Shapiro-Wilk normality tests. Comparison of the interkinetochore distance between groups was performed using an unpaired T-test. Dichotomous data, such as the percentage of aneuploid oocytes, was analyzed using Fisher’s exact test.

## Supplementary Material

Supplementary Figure 1
