# Supplementary material for: The horse as a natural model to study reproductive aging-induced aneuploidy and weakened centromeric cohesion in oocytes
Source: Aging (Albany NY). 2020 Nov 2;12(21):22220–32. doi: 10.18632/aging.104159 (PMC7695376; doi:10.18632/aging.104159)
Supplement: Supplementary Figure 1 [file aging-12-104159-s001..pdf]

SUPPLEMENTARY FIGURE

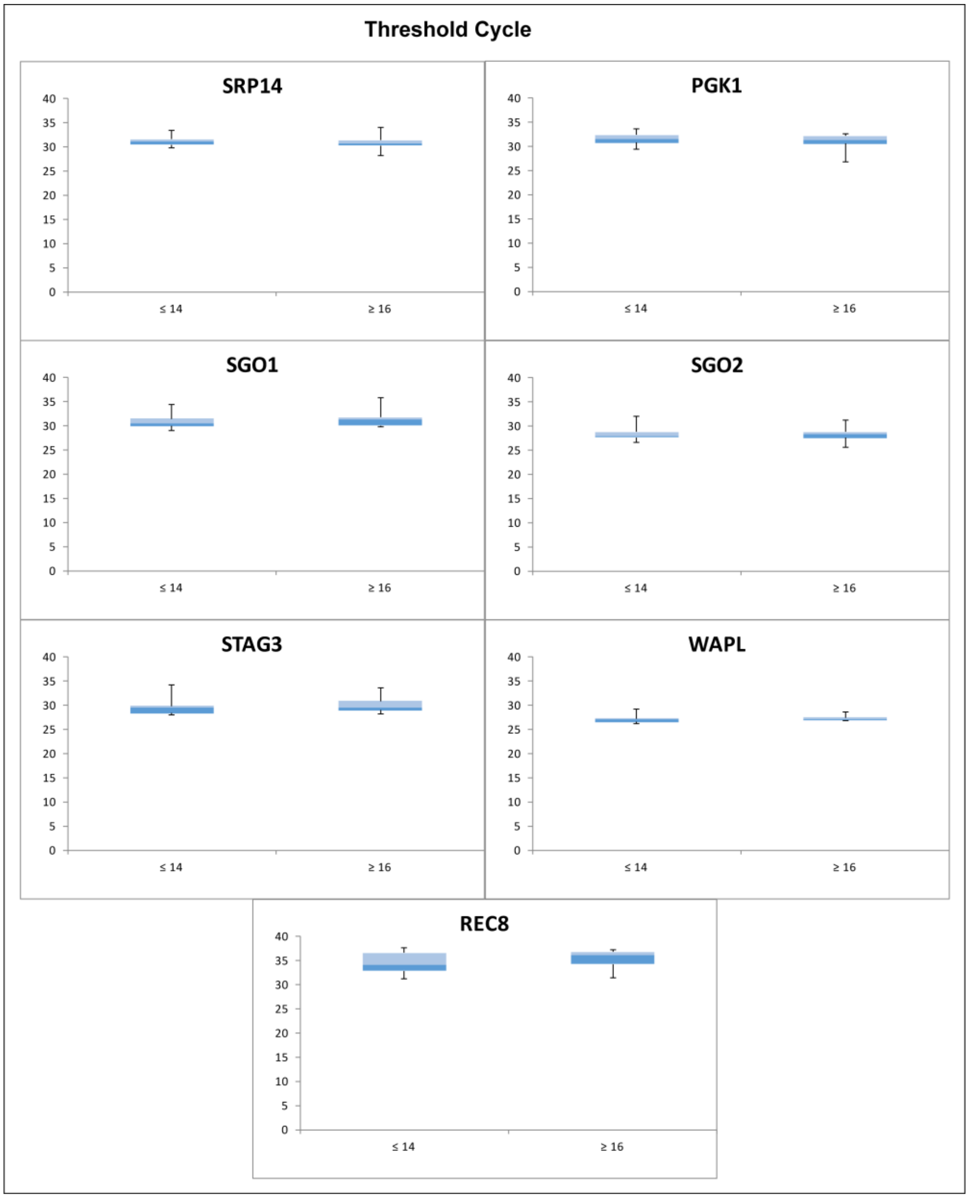

Supplementary Figure 1. Box Plot showing cycle threshold (Ct) values for all genes studied.
